# Timing of Breastfeeding Initiation Mediates the Association between Delivery Mode, Source of Breastfeeding Education, and Postpartum Depression Symptoms

**DOI:** 10.3390/nu14142959

**Published:** 2022-07-19

**Authors:** Xinran Shen, Shunna Lin, Hui Li, Nubiya Amaerjiang, Wen Shu, Menglong Li, Huidi Xiao, Sofia Segura-Pérez, Rafael Pérez-Escamilla, Xin Fan, Yifei Hu

**Affiliations:** 1 Department of Child and Adolescent Health and Maternal Care, School of Public Health, Capital Medical University, No. 10 You’anmenwai Xitoutiao, Fengtai District, Beijing 100069, China; xinranshen@163.com (X.S.); betty20000602@163.com (H.L.); 13693617970@163.com (N.A.); nmg_sw026@163.com (W.S.); limenglong_ph@163.com (M.L.); xhd19988023@163.com (H.X.); 2 Department of Pediatrics, Tianhe District Maternal and Child Hospital of Guangzhou, Guangzhou 510620, China; linshunna2002@163.com; 3 Chief Program Officer, Hispanic Health Council, 175 Main St., Hartford, CT 06106, USA; sofias@hispanichealthcouncil.org; 4 Yale School of Public Health, Yale University, New Haven, CT 06510-3201, USA; rafael.perez-escamilla@yale.edu; 5 Department of Pediatrics, Women and Children’s Hospital of Chongqing Medical University, No. 120, Longshan Road, Yubei District, Chongqin 400042, China

**Keywords:** postpartum depression, early initiation of breastfeeding, caesarean section, breastfeeding, maternity services, health care system, China

## Abstract

**Background:** Emergency cesarean section (EMCS) and breastfeeding difficulties increase the risk of postpartum depressive (PPD) symptoms. Early initiation of breastfeeding (EIBF) may not only alleviate PPD symptoms but also facilitate subsequent breastfeeding success. EMCS is a risk factor for not practicing EIBF. Therefore, it is important to understand the relationship between EMCS, EIBF, and PPD symptoms. **Methods:** We conducted a prospective cohort study in three areas of China. At baseline, a total of 965 mothers completed electronic questionnaires within 72 h postpartum. Women were screened for PPD symptoms using the Edinburgh Postpartum Depression Scale (EPDS). Multivariate logistic regression was used to identify the determinants of PPD symptoms. Mediation analysis was used to determine if EIBF mediated the relationship between delivery mode or breastfeeding education source and PPD symptoms. **Results:** The prevalence of EIBF was 40.6%; 14% of 965 mothers experienced EMCS, and 20.4% had PPD symptoms. The risk factors for developing PPD symptoms were excessive gestational weight gain (adjusted odds ratio [aOR] = 1.55, confidence interval [95% CI]: 1.03–2.33, *p* = 0.037) and EMCS (aOR = 2.05, 95% CI: 1.30–3.25, *p* = 0.002). The protective factors for developing PPD symptoms were monthly household income over CNY 10000 (aOR = 0.68, 95% CI: 0.47–0.97, *p* = 0.034), EIBF (aOR = 0.49, 95% CI: 0.34–0.72, *p* < 0.001), and prenatal breastfeeding education from nurses (aOR = 0.46, 95% CI: 0.29–0.73, *p* = 0.001). EIBF indirectly affected PPD symptoms in patients who had undergone EMCS (percentage mediated [*PM*] = 16.69, 95% CI: 7.85–25.25, *p* < 0.001). The source of breastfeeding education through EIBF also affected PPD symptoms (*PM* = 17.29, 95% CI: 3.80–30.78, *p* = 0.012). **Conclusion:** The association between EMCS on PPD symptoms was mediated by EIBF. By providing breastfeeding education, nurses could also help alleviate PPD symptoms.

## 1. Introduction

Postpartum depression (PPD) is a major public health concern, as it affects up to 10–20% of women [1] and can increase the risk of perinatal suicide [2], poor child neurocognitive development [3], and depression of the offspring in adulthood [4]. The definition of PPD remains unclear and controversial [5]. So far, depression with peripartum onset is defined in the Diagnostic and Statistical Manual of Mental Disorders, fifth edition (DSM-5) as depression that develops during pregnancy and for up to four weeks after delivery [6]. Although PPD is a significant risk factor for poor physical and mental health of both mothers and their offspring, it remains underdiagnosed and undertreated [7]. Self-administered scales designed to detect PPD symptoms have been found to adequately predict the prevalence of PPD or its risk during the early postpartum period [8,9]. Thus, screening mothers for PPD symptoms and identifying modifiable risk factors for PPD during the perinatal period has important public health implications for the well-being of mothers and their offspring.

PPD risk is exacerbated by traumatic birth experiences and inadequate social support [4]. Studies have shown that emergency caesarean section (EMCS) increases the risk of developing PPD even more than elective caesarean section (c-section) does [10,11]. This is not surprising given that elective c-section is usually planned at 37–38 weeks of pregnancy, while EMCS is the result of sudden and serious complications during pregnancy or labor [12], hence leading to greater maternal stress and pain [11]. However, it would be difficult to reduce the prevalence of PPD symptoms through the direct reduction of the EMCS rate, since the estimated prevalence of c-sections will continue to rise in this decade across the globe and is expected to reach 29% by 2030 [13], which is much higher than the WHO’s goal of keeping c-section prevalence no greater than 10–15% [14].

It is reasonable to hypothesize that the adverse effect of EMCS on PPD can be mediated through practicing early initiation of breastfeeding (EIBF), since c-section is also a risk factor for delayed breastfeeding initiation [15], and breastfeeding has been associated with a reduced risk of developing PPD [16]. EIBF prevalence is defined by the World Health Organization (WHO) and the United Nations Children’s Fund (UNICEF) as the percentage of children born in the last 24 months who were put to the breast within one hour of birth [17]. EIBF has been associated with longer breastfeeding duration [18] and higher exclusive breastfeeding (EBF) rates [19]. EIBF has also been shown to reduce morbidity and mortality in the neonatal and early infancy periods [20]. Therefore, improving EIBF with breastfeeding education as social support would not only reduce PPD symptoms but also promote breastfeeding success and improve the health of both mothers and children. This, in turn, can have strong implications for understanding if and how breastfeeding protection, promotion, and support programs, such as the Baby Friendly Hospital Initiative (BFHI), that include EIBF and breastfeeding education and support may reduce PPD symptoms [21].

Our study was designed to test the following hypotheses: (1) EMCS is independently associated with PPD symptoms; (2) the association between EMCS and PPD symptoms is mediated through EIBF; and (3) prenatal breastfeeding education from professionals may reduce PPD symptoms by increasing the prevalence of EIBF. Testing these hypotheses is key to informing the design of maternity services-based interventions seeking to reduce PPD symptoms, taking into account delivery mode.

## 2. Methods

### 2.1. Study Setting and Participants Enrollment Criteria

This study was based on a recently completed baseline survey of a multi-center prospective cohort in three regions of China (Chongqing, Guangzhou, Huizhou), examining the factors influencing breastfeeding and the cognitive–behavioral development of children from birth to 12 months after delivery (detailed elsewhere [22]). In brief, mothers were enrolled if they: (1) were between 18 and 50 years of age, (2) delivered a full-term singleton, and (3) they lived and were planning to continue to live in a local neighborhood for at least 1 year. Women were excluded from the cohort if they: (1) had intellectual challenges making it difficult to understand the questionnaire content or unable to complete the self-administrated questionnaire; (2) had a newborn who was preterm at birth; or (3) had a newborn with a critical illness such as cardiopulmonary insufficiency.

For the analyses presented in this article, we calculated the sample size based on the formula $n=\frac{Z_{1-\alpha /2}^{2}\times pq}{d^{2}}$. According to a study conducted in China in 2019 that measured PPD symptoms with the EPDS using a cutoff value of 9, the prevalence of PPD (estimated *p*) was 18.6% [23]. Based on an allowable deviation of 0.037, a statistical power of 90%, and a two-tailed significance level of 5%, we estimated a minimum sample size of 438 participants which is below the actual sample size included (n = 965), indicating that our analyses had sufficient statistical power.

From July 2019 to January 2022, 996 respondents completed the questionnaires. Our final sample size was 965 participants after excluding 31 mothers with preterm births from urban areas in the Chongqing (center 1) and Guangzhou (center 2) regions and a mixture of urban and rural areas in Huizhou’s region (center 3–5). Due to the COVID-19 pandemic, the enrollment was delayed for over one year in relation to the original protocol.

### 2.2. Outcomes

The primary outcome of this study was postpartum depression (PPD) symptoms [21,24,25,26,27]. We applied the Chinese version of the Edinburgh Postpartum Depression Scale (EPDS) to screen for PPD symptoms in mothers within 72 h postpartum [28], since it has been shown that depressive symptoms detected at 2–3 days postpartum, using EPDS with 9 as the cutoff value, can predict depression at 4–6 weeks postpartum [8,9]. The EPDS used included 10 items rated by mothers using a 4-point response option (from 0 to 3), hence the total score could range from 0 to 30, with a higher score indicating a higher risk of PPD. As recommended [28], we used the cut-off value of 9 to indicate if the mother had PPD symptoms and was at high risk of PPD (EPDS ≥ 9).

### 2.3. Exposures

The delivery mode was surveyed in the self-administrated baseline questionnaire with the question ‘Which delivery mode did you have’? Response options were natural labor, induced labor, elective c-section, and emergency c-section (EMCS). Elective c-section was defined for the mothers as occurring before the onset of labor and was planned at 37–38 weeks during pregnancy. EMCS occurred after the onset of labor and was the result of severe complications during pregnancy or delivery [12]. Before mothers could submit their responses via the online system, local doctors had to verify the accuracy of key variables, such as delivery mode, and enter the child’s anthropometric values. Since only 2 participants chose induced labor, we classified natural labor and induced labor as vaginal delivery (VD).

Women were asked to respond retrospectively about sources of prenatal breastfeeding education. They were specifically asked to indicate the different health professionals from whom they received breastfeeding information or counseling. The question was stated as ‘Did you access breastfeeding education from a professional during your pregnancy? If yes, from whom’? The response options were: (a) doctors; (b) nurses; (c) lactation specialists, (d) community health workers, (e) others, and (f) did not acquire breastfeeding education from any professional. We further classified lactation specialists and community health workers as ‘others’ in the analysis due to the limited number of participants who chose these two options.

### 2.4. Mediator

The mediator variable was timing of breastfeeding initiation which was classified as a binary variable (≤1 h vs. >1 h (ref.)) estimated from the question ‘how long after delivery did you start breastfeeding’? The corresponding response options were: ≤1 h; 1–6 h; 7–12 h; 13–24 h; >24 h after delivery; and never breastfeed. EIBF referred to the initiation of breastfeeding within 1 h after delivery, as recommended by the WHO, i.e., percentage of children born in the last 24 months who were put to breast within 1 h of birth [29]. Delayed breastfeeding initiation (DIBF) referred to the initiation of breastfeeding beyond 1 h of birth.

### 2.5. Covariates

The baseline questionnaire consisted of 50 questions of which 14 questions were selected to provide covariates for this study. Socio-demographic characteristics included maternal age, marital status, region, enrolled time, maternal educational attainment, monthly household income, and maternity leave. Self-reported biomedical maternal characteristics included pre-pregnancy BMI (undernourished, <18.5 km/m^2^; normal, 18.5–24.9 km/m^2^; overweight, 25.0–29.9 km/m^2^; obesity, ≥30 km/m^2^), parity, and gestational weight gain (GWG). GWG was defined as the difference between the maternal self-reported final weight and pre-pregnancy weight and was categorized into suboptimal GWG, appropriate GWG, and excessive GWG according to the IOM guidelines [30]. Newborn-related characteristics included the newborn’s sex and whether or not the child’s sex met the parents’ expectations.

### 2.6. Statistical Analysis

Descriptive statistics were generated using counts and percentages to describe categorical variables. Univariate logistic regression was used to explore the relationship between the outcome and exposure variables or covariates. Binary logistic regression models incorporating conceptual hierarchical frameworks were generated to explore the association between variables and PPD symptoms. Our analyses included three multivariable logistic regression models with adequate goodness of fit. Model 1 entered the covariates and delivery mode. Then, the timing of breastfeeding initiation was entered in model 2. Source of prenatal breastfeeding education was entered in model 3. Odds ratios (OR) and their 95% confidence intervals (CI) were generated by Logistic regression.

**Figure 1:**
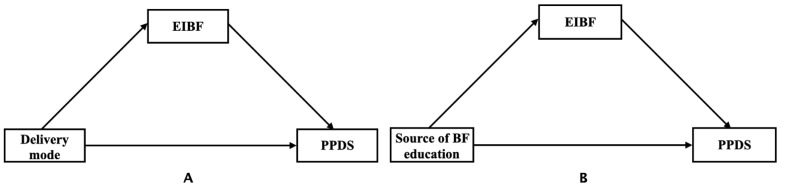
Path diagrams of mediation models examining the mediating effect of Early Initiation of Breastfeeding (EIBF) on the relationships between delivery mode (**A**), source of breastfeeding education (**B**), and postpartum depressive symptoms (PPDS). *n* = 965 Chinese mothers.

The mediation analysis was conducted to estimate the potential mediation effect of EIBF on the association between delivery mode, source of breastfeeding knowledge, and PPD symptoms (Figure 1), using the SAS PROC CAUSALMED procedure. Logistic regression was conducted to examine whether there was an interaction between exposures (delivery mode and source of breastfeeding education) and EIBF, and if the interaction was not significant on PPD symptoms (Supplementary Table S1), the interaction option would not be chosen in the PROC CAUSALMED procedure. Instead, the option would be selected. Since the occurrence of PPD symptoms is not a rare event, the binary outcome variable and mediator were both modeled by using a log link [31,32]. The exposures which are multi-categorized variables were dichotomized as EMCS vs. VD, elective c-section vs. VD, doctors vs. none, nurses vs. none, and others vs. none using PROC CAUSALMED. The total effect was defined as the difference between the counterfactual outcomes when exposures (i.e., delivery mode; source of breastfeeding education) were present (value = 1) or absent (value = 0). The natural direct effect (NDE) referred to the direct effect of exposures on PPD symptoms which was not associated with EIBF. The natural indirect effect (NIE) represented the indirect effect of exposures on PPD symptoms through EIBF. Percentage mediated (PM) represented the proportion of NIE in relation to the total effect [33]. Sensitivity analysis, excluding 11 participants with antenatal depression history, was conducted to test the robustness of our findings (Supplementary Table S2). Risk ratios (RR) and their 95% confidence intervals (CI) were generated in mediation analysis.

All statistical analyses were conducted using Statistical Analysis System V9.4 (SAS Institute Inc., Cary, NC, USA). A two-tailed *p* < 0.05 was considered as statistically significant.

## 3. Results

Overall, the prevalence of PPD symptoms was 20.4%, and 40.6% of participants had an EIBF (breastfeeding ≤ 1 h after delivery) among 965 mothers. The prevalence of vaginal delivery, cesarean sections, and EMCS were 58.3%, 41.7%, and 13.9%, respectively. Nearly 80% of mothers were aged 26–35 years, and the vast majority lived with their partners (99.8%). Most participants were enrolled in Chongqing (82.7%), followed by Guangzhou (14.8%) and Huizhou (2.5%). Approximately 90% of participants were enrolled in the cohort in 2021 once some COVID-19 epidemic restrictions were lifted. Over half of the mothers had paid maternity leave for 4–6 months, nearly 5% had no maternity leave, and 24.2% were not employed. Of the mothers, 80% had a bachelor’s degree or higher, and only 40% had a monthly household income of CNY 10,000 (equivalent to over USD 1578). In addition, 73.7% of mothers reported a pre-pregnancy BMI within the normal range, about 16% were undernourished before pregnancy, 53% gained weight during pregnancy within the IOM recommended ranges, and over 24% had an excessive GWG. Regarding health conditions, 19.6% had gestational diabetes, and 1.7% had gestational hypertension (Table 1).

As shown in Table 2, the univariate analysis indicated that mothers whose monthly household income was more than CNY 10,000 (equivalent to over USD 1578)(OR = 0.62, 95% CI: 0.44–0.86, *p* = 0.004), practiced EIBF (OR = 0.41, 95% CI: 0.29–0.58, *p* < 0.001), and accessed breastfeeding education from nurses (OR = 0.41, 95% CI: 0.27–0.63, *p* < 0.001) were less likely to have PPD symptoms, while mothers with excessive GWG (OR = 1.46, 95% CI: 1.01–2.10, *p* = 0.046) and EMCS (OR = 2.04, 95% CI: 1.33–3.13, *p* = 0.001) were more likely to have PPD symptoms. Meanwhile, no associations were observed between PPD symptoms and breastfeeding education from doctors, pre-pregnancy BMI, elective c-section, parity, and maternal educational attainment.

The results of the multivariate hierarchical model showed that a monthly household income more than CNY 10,000 (equivalent to over USD 1578) was associated with a 32% decreased risk in having PPD symptoms (aOR = 0.68, 95% CI: 0.47–0.97, *p* = 0.034; model 3). The association between excessive GWG and PPD risk in model 1 was not significant (aOR = 1.43, 95% CI: 0.96–2.13, *p* = 0.077; model 1). After adjusting for EIBF and the source of breastfeeding education, mothers with excessive GWG were more likely to have PPD symptoms than those whose GWG was appropriate (aOR = 1.55, 95% CI: 1.03–2.33, *p* = 0.037; model 3). Mothers who underwent EMCS were more likely to have PPD symptoms compared with those who had VD (aOR = 2.05, 95% CI: 1.30–3.25, *p* = 0.002; model 3). Mothers who practiced EIBF had a 51% decreased risk of developing PPD symptoms (aOR = 0.49, 95% CI: 0.34–0.72, *p* < 0.001; model 3). In addition, mothers who accessed breastfeeding education from nurses during pregnancy were less likely to have PPD symptoms than those who did not access breastfeeding education from any professionals (aOR = 0.46, 95% CI: 0.29–0.73, *p* = 0.001; model 3). There was no relationship between doctors’ provision of breastfeeding education and PPD symptoms. Multiparity, suboptimal GWG, and elective c-section were not associated with PPD symptoms (Table 3).

Logistic regression showed that the interaction between EMCS and EIBF on PPD symptoms was not significant, while the interaction between the source of breastfeeding education and EIBF was marginally significant (Supplementary Table S1). The mediation effects of EIBF between EMCS, breastfeeding education from nurses, and PPD symptoms are presented in Table 4. EMCS was associated with EIBF and the associations of EMCS with PPD symptoms were mediated through EIBF. There was a direct effect of EMCS on PPD symptoms (RR = 2.53, 95% CI: 2.04–3.02, *p* < 0.001) and an indirect effect between EMCS and PPD symptoms through EIBF (RR = 1.12, 95% CI: 1.05–1.20, *p* = 0.002). EIBF was found to be a statistically significant mediator between EMCS and PPD symptoms (*PM* = 16.69, 95% CI: 7.85–25.52, *p* < 0.001).

Furthermore, the indirect effects of breastfeeding education from nurses on PPD symptoms through EIBF were significant (RR = 0.80, 95% CI: 0.70–0.91, *p* < 0.001), and the direct effects of breastfeeding education from nurses on PPD symptoms were also significant (RR = 0.51, 95% CI: 0.34–0.69, *p* < 0.001). Only 17.29% of nurses’ effects were attributed to the mediation of EIBF (*PM* = 17.29, 95% CI: 3.80–30.78, *p* = 0.012). No significant mediating role of EIBF was found between breastfeeding education from doctors or elective c-sections and PPD symptoms.

Sensitivity analyses conducted by excluding 11 participants with prenatal depression yielded similar findings when compared to the analysis with the whole analytical sample (Supplementary Table S2).

## 4. Discussion

To our knowledge, this is the first study to explore the association between emergency caesarean section (EMCS) and the risk of postpartum depression (PPD) mediated through early initiation of breastfeeding (EIBF). Our results demonstrated that EMCS and excessive gestational weight gain (GWG) increased the risk of developing PPD symptoms, while EIBF and breastfeeding education accessed from nurses were protective factors from developing PPD symptoms. EIBF could alleviate to some extent the relationship between EMCS and PPD symptoms. Moreover, the risk of PPD could be further modified by the influence breastfeeding education from nurses during pregnancy had on EIBF.

The overall prevalence of PPD symptoms in the present study was 20.4%, which is similar to the 20% reported by a systematic review study in low and middle income countries [34] and higher than the 13% reported by a study in Guangzhou, using the EPDS with a cutoff value of 13 [35]. This may be explained by the differences in the timing of PPD measurements, measurement instruments, and the choice of cutoff values across studies. To be specific, measuring symptoms at 3 days postpartum in this study may overestimate the risk of PPD due to the presence of postpartum blues. Postpartum blues is a transient emotional instability caused by hormonal fluctuations, peaking 3–5 days after delivery, and can last for days or even weeks. Since the symptoms of postpartum blues are similar to PPD [4], postpartum measurement may lead to an overestimation of the risk of PPD. The present study demonstrated an overall EIBF rate of 40.6%, which corresponds to the third out of four categories. Specifically, WHO uses color codes for assessing EIBF levels: green (≥70%), yellow (50–70%), orange (30–50%), and red (<30%) [36]. The EIBF rating found in our study can be compared to a national survey in China in 2013 (28.7%) [37] and another survey in 12 Chinese provinces conducted in 2017–2018 (8.2%) [38]. The EIBF fluctuations in China may be explained by differences in sampling strategies and socio-economic status across studies. Moreover, in this study, the EIBF rate may have been overestimated since mothers of preterm babies were excluded.

Excessive GWG was associated with a 55% risk increase for developing PPD symptoms compared to appropriate GWG, albeit no association was found between suboptimal GWG and PPD symptoms. Excessive GWG may foster a negative body image in women, and this could partially explain its association with PPD symptoms [25]. So far, there was no consistent opinion on the relationship between GWG and PPD symptoms, and a meta-analysis including 16 studies showed that excessive and suboptimal GWG were both significantly associated with a higher risk of PPD development [39]. However, a study in Japan showed that the association between excessive GWG and PPD symptoms diminished in the subgroup analysis by pre-pregnancy BMI [25]. Nevertheless, it is still recommended controlling GWG during pregnancy with exercise and healthy dietary patterns [40] to reduce PPD risk. Meanwhile, professionals should pay more attention to screen PPD symptoms among mothers with inadequate GWG.

Our study confirmed the hypothesis that EMCS was associated with PPD symptoms. Multivariate analyses showed that mothers who experienced EMCS were 1.05 times more likely to be at risk of PPD than those who had vaginal delivery, while the association between elective c-section and PPD symptoms was not significant. This finding is similar to the results of a meta-analysis, where c-section was a risk factor for PPD compared to vaginal delivery, and EMCS was more likely to cause mild PPD than elective c-sections [11]. The difference in the association between EMCS versus elective c-section and PPD may be due to several reasons. First, mothers who underwent EMCS may have suffered more prolonged and unexpected psychological and physical trauma than mothers who underwent elective c-sections [11]. Second, the negative birth experience of EMCS may increase risk of PPD through PTSD symptoms [12]. Third, general anesthetics commonly used in EMCS are more likely to cause PPD than spinal anesthesia used in elective c-sections [41]. Thus, reducing the rate of unnecessary EMCS is critical for mitigating PPD. The guidelines for performing EMCS have not been standardized and should be further refined [42]. Moreover, the factors leading to the switch from vaginal delivery to EMCS should be taken into account when developing guidelines for elective c-sections [43], and the indication for elective c-sections should be evidence-based to reduce the frequency of EMCS. Moreover, since EMCS relies heavily on physicians’ decision making at the time [43], it is important to improve the standard of primary care in all regions and corresponding trainings of health care professionals.

Our second hypothesis was also verified as approximately 16.7% of the effect of EMCS on PPD risk was mitigated by EIBF. In other words, EMCS may exacerbate PPD symptoms by delaying the timing of breastfeeding initiation. Several previous studies have shown that c-section is a risk factor for not practicing EIBF [15,38]. A study in Dubai reported that EMCS was associated with a 14% increased risk of delayed breastfeeding initiation compared to elective c-section [44]. This may be due to a more stressful birth experience preventing maternal or health care system readiness for EIBF [15]. In our study, EIBF partially mediated the association of EMCS with PPD symptoms, and multivariate analysis also showed that EIBF reduced the risk of developing PPD symptoms by 51%. A previous study conducted in the United States showed that mothers who experienced difficulties during early breastfeeding were more likely to have PPD symptoms [45]. It is possible that effective suckling from the nipple prompts the abundant release of prolactin, which in turn stimulates breast milk production [46], reduces breastfeeding difficulties, and as a result may reduce the risk of PPD. In addition, skin-to-skin contact elevates oxytocin levels and thus may improve maternal mood [47]. To sum up, EIBF is the foundation for subsequent breastfeeding success [19], and successful breastfeeding could further reduce the risk of PPD [16]. Hence it is important to adjust all maternity hospital settings to facilitate EIBF by making it a very easy choice for the mother. Enhancing EIBF through maternity services-based interventions would indeed be a cost-effective approach to improve postpartum mood and could help alleviate the influence of EMCS on PPD risk, at least to some extent [48].

This study also examined the mediating role of EIBF on the association between sources of breastfeeding education during pregnancy and PPD symptoms. Mothers who had access to breastfeeding education from a nurse were 54% less likely to have PPD symptoms than mothers who did not access education from any professionals. Such was not found when the breastfeeding education source was a doctor or other health care personnel. This is because nurses are often the ones who introduce breastfeeding information to pregnant women and are available to them during the immediate period after birth, compared to other professionals [49]. In addition, nurses spend more time with patients than doctors and are more ready to communicate and educate with empathy.

Our third hypothesis was also partially validated. The mediation model found that breastfeeding education from nurses explained 82.71% of the direct effect on improving PPD symptoms, while the remaining 17.29% was explained through indirect effects. In other words, breastfeeding education from nurses was associated with reduced risk of PPD in women only partly through practicing EIBF, but mainly through other pathways that were not elucidated in this study. Consistent with this interpretation, a previous study found that the Baby-Friendly Hospital Initiative (BFHI) improved depressive symptoms through pathways other than enhanced breastfeeding outcomes [21]. BFHI may improve postpartum mood by enhancing prenatal breastfeeding information and increasing breastfeeding self-efficacy, rather than solely by improving breastfeeding behaviors [50]. Moreover, social support from professionals may mitigate PPD symptoms by reducing the impact of birth-related and other stressors [51]. Therefore, breastfeeding-related interventions such as effective breastfeeding education by health care professionals may help mitigate PPD symptoms. This may be particularly important in many countries, including China, where maternity-related psycho-emotional interventions are not widely available. Prospective intervention research is needed to better understand how to design, implement, and maintain breastfeeding support interventions that help mitigate the risk of PPD on a large scale. Such studies need to consider the potential bidirectional relationship between breastfeeding and PPD symptoms; i.e., breastfeeding interventions may reduce the risk of PPD at the same time that PPD risk reduction interventions may improve breastfeeding outcomes.

## 5. Limitations

Our study has several limitations. First, the mediation analyses were performed with the baseline survey of the ongoing cohort study which is cross-sectional in nature, potentially affecting the temporal sequence of events in our mediation models. However, it is important to note that the temporal sequence of data collection was appropriate to test our hypotheses as EIBF occurs within 1 h postpartum while PPD symptoms were assessed at 3 days postpartum. Second, screening symptoms of PPD at 3 days postpartum may overestimate the prevalence of PPD symptoms as they capture a mixture of prenatal depression, postpartum blues and depression [6]. The boundaries between PPD and postpartum blues are indeed difficult to delineate [5] since postpartum blues peaking 3–5 days after delivery have similar symptoms to PPD [4]. However, previous studies have shown that EPDS scores at 2–3 days postpartum can predict depressive symptoms at 4–6 weeks postpartum [8,9], and testing mothers before being discharged from the hospital provides an opportunity to screen people at high risk of PPD in a timely and more comprehensive way [52]. Thus, while designing the study, we concluded that to minimize missing data on PPD symptoms data due to loss to follow-up, applying the EPDS before hospital discharge was the most reasonable option, especially because it was supported by a very robust study that involved a direct correlation of PPD symptoms assessed at day 2 postpartum with a psychiatric diagnosis of PPD three to four weeks postpartum [8]. Moreover, although the effect of prenatal depression on PPD risk was not included in the models, the confounding effect of prenatal depression on PPD symptoms was limited according to the consistent result of the sensitivity analysis that removed the few participants who declared they had prenatal depression.

## 6. Conclusions

This study provided evidence that EMCS was negatively associated with PPD symptoms, and the association was partially mediated by EIBF. Therefore, the maternity services-based interventions that increase the prevalence of EIBF could reduce the occurrence of PPD symptoms to some extent. Breastfeeding education from nurses during pregnancy was found to be a protective factor for developing PPD symptoms. These findings suggested nurses could be mobilized for improving the coverage and quality of breastfeeding counseling services to both improve breastfeeding outcomes and reduce the risk of PPD.

## Figures and Tables

**Table 1:** Descriptive characteristics of Chinese women and their infants, categorized by the risk of developing PPD.

Characteristics	nPPD (*n* = 768)	PPD (*n* = 197)	Total (*n* = 965)
Maternal age (years)			
≥36	62 (11.6)	21 (13.7)	83 (12.0)
26–35	617 (80.3)	149 (75.6)	766 (79.4)
18–25	89 (8.1)	27 (10.7)	116 (8.6)
Marital status			
Married/Living with a partner	767 (99.9)	196 (99.5)	963 (99.8)
Single/Divorced/Widowed	1 (0.1)	1 (0.5)	2 (0.2)
Region			
Huizhou	17 (2.2)	7 (3.6)	24 (2.5)
Chongqing	647 (84.2)	151 (76.7)	798 (82.7)
Guangzhou	104 (13.5)	39 (19.8)	143 (14.8)
Enrolled time			
2019	67 (8.7)	18 (9.1)	85 (8.8)
2020	3 (0.4)	0 (0.0)	3 (0.3)
2021	698 (90.9)	179 (90.9)	877 (90.9)
Maternity leave			
None	37 (4.8)	8 (4.1)	45 (4.7)
≤3 months	56 (7.3)	16 (8.1)	72 (7.5)
4–6 months	457 (59.5)	106 (53.8)	563 (58.3)
≥7 months	42 (5.5)	10 (5.1)	52 (5.4)
Unemployed	176 (22.9)	57 (28.9)	233 (24.2)
Monthly household income (USD)			
≤1262	149 (19.4)	58 (29.4)	207 (21.5)
1262–1578	295 (38.4)	78 (39.6)	373 (38.7)
≥1578	324 (42.2)	61 (31.0)	385 (39.9)
Maternal educational attainment			
Primary School or below	2 (0.3)	1 (0.5)	3 (0.3)
Junior High School	35 (4.6)	14 (7.1)	49 (5.1)
High school	119 (15.5)	34 (17.3)	153 (15.9)
Bachelor’s degree or higher	612 (79.7)	148 (75.1)	760 (78.8)
BMI of mother			
Undernourished	122 (15.9)	33 (16.8)	155 (16.1)
Normal	567 (73.8)	144 (73.1)	711 (73.7)
Overweight	62 (8.1)	13 (6.6)	75 (7.8)
Obesity	17 (2.2)	7 (3.6)	24 (2.5)
Gestational weight gain			
Appropriate GWG	419 (54.6)	96 (48.7)	515 (53.4)
Excessive GWG	177 (23.1)	59 (30.0)	236 (24.5)
Suboptimal GWG	172 (22.4)	42 (21.3)	214 (22.2)
Gestational diabetes			
No	614 (80.0)	162 (82.2)	776 (80.4)
Yes	154 (20.1)	35 (17.8)	189 (19.6)
Gestational hypertension			
No	756 (98.4)	193 (98.0)	949 (98.3)
Yes	12 (1.6)	4 (2.0)	16 (1.7)
Delivery mode			
Vaginal delivery	463 (60.3)	100 (50.8)	563 (58.3)
Elective C-section	212 (27.6)	56 (28.4)	268 (27.8)
Emergency C-section	93 (12.1)	41 (20.8)	134 (13.9)
Sex of baby			
Male	410 (53.4)	95 (48.2)	505 (52.3)
Female	358 (46.6)	102 (51.8)	460 (47.7)
Sex expectation ^a^			
Matched	737 (96.0)	184 (93.4)	921 (95.4)
Did not match	31 (4.0)	13 (6.6)	44 (4.6)
Parity			
1	520 (67.7)	135 (68.5)	655 (67.9)
2	234 (30.5)	60 (30.5)	294 (30.5)
≥3	14 (1.8)	2 (1.0)	16 (1.7)
Timing of early BF initiation			
Delayed BF initiation	425 (55.3)	148 (75.1)	573 (59.4)
EIBF	343 (44.7)	49 (24.9)	392 (40.6)
Source of BF education ^b^			
None	221 (28.8)	73 (37.1)	294 (30.5)
Doctors	210 (27.3)	68 (34.5)	278 (28.8)
Nurses	266 (34.6)	36 (18.3)	302 (31.3)
Others	71 (9.2)	20 (10.2)	91 (9.4)

nPPD, mothers with PPD symptoms score below cut-off point suggested they were at no or low risk of developing PPD; PPD, mothers with PPD postpartum symptoms scores above cut-off point, suggestive of risk of PPD; C-section, cesarean delivery; GWG, gestational weight gain; BF, breastfeeding; EIBF, early initiation of breastfeeding; USD 1 ≈ CNY 6.34; ^a^: whether the child’s gender meets the parents’ expectations; ^b^: Did you access breastfeeding education from a professional during your pregnancy? If yes, from whom?

**Table 2:** Factors associated with postpartum depressive symptoms score above the cut-off point of the Edinburgh Postpartum Depression Scale (EPDS) among women in three regions in China. Univariate logistic regression model (*n* = 965).

Characteristics	OR (95% CI)	*p*
Maternal age (years)		
≥36	ref.	-
26–35	0.71 (0.42, 1.21)	0.21
18–25	0.90 (0.47, 1.73)	0.74
Region		
Huizhou	ref.	-
Chongqing	0.57 (0.23, 1.39)	0.22
Guangzhou	0.91 (0.35, 2.36)	0.85
Monthly household income (USD)		
≤1578	ref.	-
>1578	0.62 (0.44, 0.86)	0.004
Maternal BMI		
Undernourished	ref.	-
Normal	0.94 (0.61, 1.44)	0.77
Overweight/obesity	0.94 (0.50, 1.75)	0.84
Gestational weight gain		
Appropriate GWG	ref.	-
Excessive GWG	1.46 (1.01, 2.10)	0.046
Suboptimal GWG	1.07 (0.71, 1.60)	0.76
Delivery mode		
Vaginal delivery	ref.	-
Elective C-section	1.22 (0.85, 1.76)	0.28
Emergency C-section	2.04 (1.33, 3.13)	0.001
Sex of baby		
Male	ref.	-
Female	1.23 (0.90, 1.68)	0.20
Sex expectation ^a^		
Matched	ref.	-
Did not match	1.68 (0.86, 3.27)	0.13
Parity		
Primiparity	ref.	-
Multiparity	0.96 (0.69, 1.35)	0.83
Timing of BF initiation		
Delayed BF initiation	ref.	-
Early Initiation of BF (EIBF)	0.41 (0.29, 0.58)	<0.001
Source of BF education ^b^		
None	ref.	-
Doctors	0.98 (0.67, 1.43)	0.92
Nurses	0.41 (0.27, 0.63)	<0.001
Others	0.85 (0.49, 1.50)	0.58

OR: odds ratio; CI: confidence interval; GWG, gestational weight gain; BF, breastfeeding; EIBF, early initiation of breastfeeding; USD 1 ≈ CNY 6.34; a: whether the child’s gender meets the parents’ expectations; b: Did you access breastfeeding education from a professional during your pregnancy? If yes, from whom?

**Table 3:** Factors associated with postpartum depressive symptoms score above the cut-off point of the Edinburgh Postpartum Depression Scale (EPDS) among women in three regions in China. Multivariable hierarchical models (*n*= 965).

Characteristics	Model 1		Model 2		Model 3	
	aOR (95% CI)	*p*	aOR (95% CI)	*p*	aOR (95% CI)	*p*
Maternal age (years)						
≥36	ref.	-	ref.	-	ref.	-
26–35	0.68 (0.39, 1.20)	0.19	0.68 (0.39, 1.22)	0.20	0.71 (0.40, 1.26)	0.24
18–25	0.73 (0.35, 1.53)	0.40	0.79 (0.37, 1.69)	0.54	0.83 (0.39, 1.77)	0.62
Region						
Huizhou	ref.	-	ref.	-	ref.	-
Chongqing	0.49 (0.14, 1.71)	0.26	0.54 (0.16, 1.88)	0.33	0.58 (0.16, 2.05)	0.40
Guangzhou	0.79 (0.22, 2.83)	0.72	0.68 (0.19, 2.42)	0.55	0.77 (0.21, 2.78)	0.69
Monthly household income (USD)						
≤1578	ref.	-	ref.	-	ref.	-
>1578	0.65 (0.46, 0.93)	0.019	0.67 (0.47, 0.96)	0.031	0.68 (0.47, 0.97)	0.034
BMI of mother						
Undernourished	ref.	-	ref.	-	ref.	-
Normal	0.98 (0.62, 1.52)	0.91	1.01 (0.64, 1.58)	0.98	0.96 (0.61, 1.51)	0.85
Overweight/obesity	0.67 (0.34, 1.32)	0.25	0.66 (0.33, 1.32)	0.24	0.60 (0.30, 1.20)	0.15
Gestational weight gain						
Appropriate GWG	ref.	-	ref.	-	ref.	-
Excessive GWG	1.43 (0.96, 2.13)	0.077	1.48 (0.99, 2.21)	0.058	1.55 (1.03, 2.33)	0.037
Suboptimal GWG	1.00 (0.65, 1.52)	0.99	1.01 (0.66, 1.55)	0.96	1.02 (0.67, 1.57)	0.92
Delivery mode						
Vaginal delivery	ref.	-	ref.	-	ref.	-
Elective C-section	1.35 (0.91, 2.01)	0.14	1.29 (0.86, 1.94)	0.22	1.35 (0.90, 2.04)	0.15
Emergency C-section	2.32 (1.48, 3.64)	<0.001	2.10 (1.33, 3.32)	0.001	2.05 (1.30, 3.25)	0.002
Sex expectation ^a^						
Matched	ref.	-	ref.	-	ref.	-
Did not match	1.76 (0.87, 3.54)	0.11	1.70 (0.84, 3.43)	0.14	1.63 (0.80, 3.32)	0.18
Parity						
Primiparity	ref.	-	ref.	-	ref.	-
Multiparity	0.94 (0.63, 1.38)	0.74	0.95 (0.64, 1.41)	0.80	0.93 (0.62, 1.38)	0.71
Timing of early BF initiation						
Delayed BF initiation			ref.	-	ref.	-
EIBF			0.44 (0.30, 0.64)	<0.001	0.49 (0.34, 0.72)	<0.001
Source of BF education ^b^						
None					ref.	-
Doctors					1.00 (0.67, 1.50)	1.00
Nurses					0.46 (0.29, 0.73)	0.001
Others					0.90 (0.50, 1.62)	0.71

All models above were adjusted for enrolled time, marital status, maternity leave, maternal educational attainment, newborn weight, newborn length, sex, and sex expectation. Model 1: entered variables of sociodemographic characteristics, mother-related characteristics (age, BMI, GWG, delivery mode, EIBF), and child-related characteristics (sex expectation, parity). Model 2: entered variables of model 1 + timing of BF initiation. Model 3: entered variables of model 2 + source of prenatal BF education. aOR: adjusted odds ratio; CI: confidence interval; GWG, gestational weight gain; BF, breastfeeding; EIBF, early initiation of breastfeeding [USD 1 ≈ CNY 6.34; ^a^: whether the child’s gender meets the parents’ expectation; ^b^: Did you access breastfeeding education from a professional during your pregnancy? If yes, from whom?

**Table 4:** Association between nurse as breastfeeding education source, EMCS, and postpartum depressive symptoms, China (*n* = 965).

Exposure	Effect	RR (95% CI)	*p*
Delivery mode			
EMCS ^a^ (*n* = 134) vs. VD ^b^ (ref, *n* = 563)			
	Total effect	2.84 (2.26, 3.42)	<0.001
	Natural direct effect	2.53 (2.04, 3.02)	<0.001
	Natural Indirect effect	1.12 (1.05, 1.20)	0.002
	Percentage mediated (%)	16.69 (7.85, 25.52)	<0.001
Source of BF education ^c^			
Nurse ^d^ (*n* = 302) vs. None ^e^ (ref, *n* = 294)			
	Total effect	0.41 (0.27, 0.55)	<0.001
	Natural direct effect	0.51 (0.34, 0.69)	<0.001
	Natural Indirect effect	0.80 (0.70, 0.91)	<0.001
	Percentage mediated (%)	17.29 (3.80, 30.78)	0.012

RR: relative risk; CI: confidence interval; ^a^: emergency caesarean section; ^b^: vaginal delivery; ^c^: The question is ‘Did you access breastfeeding education from a professional during your pregnancy? If yes, from whom’? This model was adjusted for maternity leave, maternal educational attainment, monthly household income, BMI of mother, gestational weight gain, delivery mode, newborn weight, newborn length, sex, sex expectation, and parity; ^d^: accessing breastfeeding education from nurses; ^e^: no prenatal breastfeeding education accessed from any professionals. Models were adjusted for maternity leave, maternal educational attainment, monthly household income, BMI of mother, gestational weight gain, delivery mode, newborn weight, newborn length, sex, sex expectation, parity, and source of breastfeeding education.

## Data Availability

All relevant data are within the manuscript and individual data could be accessed from corresponding author upon reasonable request.

## Supplementary Materials

The following supporting information can be downloaded at: https://www.mdpi.com/article/10.3390/nu14142959/s1, Table S1: Sensitivity analysis examining the interaction between EMCS, BF education from nurses, and EIBF, China (*n* = 965); Table S2: Sensitivity analysis excluding participants with prenatal depression: association between nurse as breastfeeding knowledge source, EMCS and postpartum depressive symptoms, China (*n* = 954).
