# Supplementary material for: Timing of Breastfeeding Initiation Mediates the Association between Delivery Mode, Source of Breastfeeding Education, and Postpartum Depression Symptoms
Source: Nutrients. 2022 Jul 19;14(14):2959. doi: 10.3390/nu14142959 (PMC9324203; doi:10.3390/nu14142959)
Supplement: Supplementary file 1 [file nutrients-14-02959-s001.zip › nutrients-1777838-supplementary.pdf]

Supplementary Materials

Table S1. Sensitivity analysis examining the interaction between EMCS, BF education from nurses, and EIBF, China (n=965)

Models	Variations	*β*	SE	*p*
Model 1				
	EIBF	-0.98	0.20	<0.001
	EMCS	0.41	0.25	0.100
	ELCS	0.19	0.19	0.31
	EIBF×EMCS [a]	0.79	0.48	0.096
Model 2				
	EIBF	-0.57	0.20	0.006
	Nurses	-0.45	0.26	0.087
	Doctors	-0.14	0.29	0.64
	Others	-0.01	0.20	0.95
	EIBF×Nurses [b]	-0.91	0.45	0.044

SE, standard error; EIBF, early initiation of breastfeeding; EMCS, emergency c-section; ELCS, elective c-section; nurses, breastfeeding education from nurses; doctors, breastfeeding education from doctors; others, breastfeeding education from others;

[a]: the interaction between EIBF and EMCS;

[b]: the interaction between EIBF and Nurses.

Table S2. Sensitivity analysis excluding participants with prenatal depression: association between nurse as breastfeeding knowledge source, EMCS and postpartum depressive symptoms, China (n=954)

Exposure	effect	RR (95%CI)	*p*
Delivery mode			
EMCS [a] (n=134) vs. VD [b] (ref, n=563)			

Total effect	2.07(1.43,2.70)	0.001
Natural direct effect	1.90(1.32,2.48)	0.002
Natural Indirect effect	1.09(1.03,1.15)	0.004
Percentage mediated	15.63(5.30,25.97)	<0.001
Breastfeeding knowledge from [c]		
Nurse [d](n=302) vs. None [e] (ref, n=663)		
Total effect	0.42(0.28,0.56)	<.0001
Natural direct effect	0.53(0.36,0.69)	<0.001
Natural Indirect effect	0.80(0.69,0.90)	<0.001
Percentage mediated	18.65(5.31,31.99)	<0.001

RR: Risk Ratio, CI: Confidence Interval.
a: Emergency caesarean section.
b: Vaginal delivery.
c: The question is "Did you receive breastfeeding knowledge from a professional during your pregnancy". This model was adjusted for maternity leave, maternal educational attainment, monthly household income, BMI of mother, gestational weight gain, delivery mode, newborn weight, newborn length, sex, sex expectation, and parity.
d: Acquiring breastfeeding knowledge from nurses.
e: No knowledge acquired from any professionals.
f: Delayed initiation of breastfeeding, initiation breastfeeding beyond 1 hour after birth.
g: Early initiation of breastfeeding, initiation breastfeeding within 1 hour after birth.
Models were adjusted for maternity leave, maternal educational attainment, monthly household income, BMI of mother, gestational weight gain, delivery mode, newborn weight, newborn length, sex, sex expectation, parity, and source of breastfeeding knowledge.
